# Chrom3D: three-dimensional genome modeling from Hi-C and nuclear lamin-genome contacts

**DOI:** 10.1186/s13059-016-1146-2

**Published:** 2017-01-30

**Authors:** Jonas Paulsen, Monika Sekelja, Anja R. Oldenburg, Alice Barateau, Nolwenn Briand, Erwan Delbarre, Akshay Shah, Anita L. Sørensen, Corinne Vigouroux, Brigitte Buendia, Philippe Collas

**Affiliations:** 1 Department of Molecular Medicine, Institute of Basic Medical Sciences, Faculty of Medicine, University of Oslo, Oslo, Norway; 2 Institut BFA, Université Paris 7-CNRS, Paris, France; 3 INSERM, UMR S938, Centre de Recherches Saint-Antoine, Paris, France; 4 UPMC Université Paris 6 UMR S938, Paris, France; 5 ICAN, Paris, France; 6 AP-HP Hôpital Tenon, Paris, France; 7 Norwegian Center for Stem Cell Research, Oslo University Hospital, Oslo, Norway

**Keywords:** Three-dimensional (3D) genome, Hi-C, Lamin-associated domain (LAD), Laminopathy, Modeling, Nuclear lamin, Topologically-associated domain (TAD)

## Abstract

Current three-dimensional (3D) genome modeling platforms are limited by their inability to account for radial placement of loci in the nucleus. We present Chrom3D, a user-friendly whole-genome 3D computational modeling framework that simulates positions of topologically-associated domains (TADs) relative to each other and to the nuclear periphery. Chrom3D integrates chromosome conformation capture (Hi-C) and lamin-associated domain (LAD) datasets to generate structure ensembles that recapitulate radial distributions of TADs detected in single cells. Chrom3D reveals unexpected spatial features of LAD regulation in cells from patients with a laminopathy-causing lamin mutation. Chrom3D is freely available on github.

**Electronic supplementary material:**

The online version of this article (doi:10.1186/s13059-016-1146-2) contains supplementary material, which is available to authorized users.

## Background

Advances in molecular and computational techniques have enhanced our understanding of the three-dimensional (3D) organization of eukaryotic genomes [1]. Current interpretation of chromosome-chromosome contacts determined from genome-wide chromosome conformation capture (Hi-C) data pictures a hierarchically organized genome with fundamental ~1 Mb units termed topologically associated domains (TADs) [2–4]. In mammals, the genomic linear position of TADs and TAD boundaries are overall conserved between cell types [2, 3]. However, TADs can differ in their internal chromatin folding patterns, chromatin states, and transcriptional activity [3], and contacts between TADs can be altered during cell differentiation [5]. While these observations suggest an orchestrated genome topology [6, 7], processes modulating transcriptional activity of TADs remain largely unknown.

One way for the cell to regulate chromatin activity in TADs would be to place them in distinct nuclear compartments, such as the nuclear interior which is conducive of transcriptional activity or the nuclear periphery (NP) which provides a more repressive environment. At the NP, chromatin interacts with the nuclear lamina, a meshwork of A- and B-type nuclear lamins [8], through lamin-associated domains (LADs) [9]. While lamin B1 (abbreviated as LMNB1 here) is restricted to the NP, lamins A and C, splice variants of the *LMNA* gene (abbreviated as LMNA), also exist in the nuclear interior [10] where they seem to play a role in gene regulation and differentiation [11] presumably by interacting with chromatin [6, 7]. Thus, a dynamic association of TADs with the NP would constitute a mode of regulation of transcriptional activity within TADs [3]. However, TAD positioning in the 3D nucleus space has not been examined because there are currently no means of assessing spatial mammalian genome conformation using chromatin anchor-point information. This limits our understanding of principles of genome dynamics.

Chromatin connections with intranuclear structures such as the nuclear lamina [9] contribute to spatial genome organization and regulation of gene expression. In yeast, attachment of centromeres to the spindle pole body and tethering of telomeres to the NP [12–14] provide constraints on chromosome movement which have proven useful to generate 3D genome structures [15, 16]. These observations suggest that integrating positional constraints from various genomic datasets, such as LAD information from chromatin immunoprecipitation sequencing (ChIP-seq) of nuclear lamins, in addition to Hi-C, would provide more realistic structures of the mammalian genome.

A strategy to study genome conformation is to computationally model 3D structures of chromatin and analyze the properties of these structures. 3D genome modeling approaches have been applied at various scales and resolutions [16–33]. One approach to modeling genomes from Hi-C data is to reconstruct a consensus 3D structure, using multidimensional scaling [17, 20, 21, 34] or Bayesian inference methods such as Bayesian 3D constructor for Hi-C data (BACH) and derivatives thereof [35]. Other methods recapitulate structural variations in genome conformation across cells in a population by simulating ensembles of structures [18, 19, 24, 28, 31, 35] or by data deconvolution [22, 24, 25, 31, 36]. A commonly used framework that models ensembles of structures is the Integrative Modeling Platform (IMP) [24, 31, 36, 37] (https://3dgenomes.github.io/TADbit). However, IMP has not been designed for genome modeling and requires advanced programming skills. Another constrained optimization approach (BACH-MIX) designed for local genome modeling, relies on Bayesian inference of 3D chromosome arrangements to assess variations in genome structures in a cell population [35]. BACH-MIX, however, is not designed to incorporate positional constraints for loci in the nucleus. There is therefore no user-friendly framework that models the 3D genome over a wide range of scales and that incorporates chromosome positional constraints.

We introduce Chrom3D, a genome 3D modeling platform designed to integrate positional constraints based on association of loci with intranuclear anchors. The combination of Hi-C and LAD information enables genome-wide radial positioning of TADs in ensembles of 3D structures. We also show that Chrom3D provides new opportunities to investigate mechanisms of spatial gene regulation in diseases susceptible to affect spatial chromatin organization.

## Results

### A 3D genome modeling framework integrating chromosomal interactions and radial position information

**Fig. 1:**
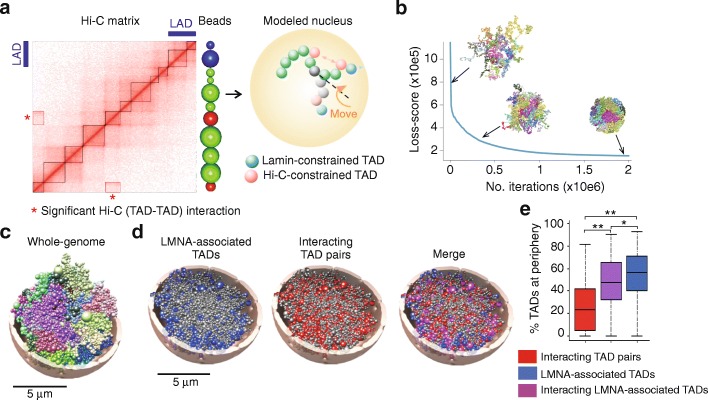
Chrom3D integrates Hi-C and nuclear lamin ChIP-seq data to provide an ensemble of 3D genome structures with radial positioning information of loci. **a** Chrom3D principles. Hi-C and lamin ChIP-seq data are combined to define beads (TADs) subjected to interaction constraints between them, based on Hi-C data, or to interaction constraints with the NP, based on LMNA ChIP-seq data (LADs). Hi-C and LAD maps shown are dummies for explanation purposes. Additional file 1: Figure S2 shows an actual representation of the relationship between TADs and LADs. During a simulation, TADs are rearranged with a modeled nucleus by a chromosome move (*orange arrow*) selected among a set of five possibilities (Additional file 1: Table S1) in order to juxtapose two interacting TADs (*red beads*), and position a LMNA-associated TAD at the NP (*blue bead*). Radius of the modeled nuclei is 5 μm. **b** Loss-score values and representative structures during a simulation; each chromosome is colored differently. **c** Example of a Chrom3D whole-genome 3D structure; chromosomes are distinctively colored. **d** Tomographic views of the structure in (**c**), showing LMNA-associated TADs (*blue beads*), all interacting TAD pairs *(red beads*), and interacting TAD pairs in which at least one TAD is associated with LMNA (*purple “merged” beads*). *Gray beads* visualize all other TADs. **e** Percentage of TADs at the NP as a function of Hi-C and LMNA constraints across 400 structures; **P < 2.2 × 10^–16^; *P = 8.53 × 10^–5^ (Mann–Whitney U tests)

Chrom3D simultaneously incorporates chromosomal interaction constraints and constraints from chromosome association with the nuclear lamina, at the NP (Fig. 1a; Additional file 1: Figure S1). Each chromosome is modeled as a beads-on-a-string chain where each bead represents a genomic contact domain (TAD). To develop Chrom3D, we integrated statistically significant pair-wise interactions between TADs (interacting TAD pairs) identified from high-resolution Hi-C data in HeLa cells [38] and association of TADs with the NP determined by ChIP-seq of LMNA also in HeLa cells [7] (Additional file 1: Figure S1). In effect, if a DNA sequence identified as a LAD can be assigned to a TAD (see “Methods”), Chrom3D will constrain this TAD to the NP; we refer to such TADs as LMNA-associated TADs (Additional file 1: Figure S1). Chrom3D therefore emphasizes constraints that are significantly enriched in the population-based Hi-C and lamin ChIP-seq data; Additional file 1: Figure S2 depicts all positional constraints for each chromosome. Instead of optimizing particular distances between a large number of bead pairs, our approach enables an emphasis on the subset of bead pairs that significantly interact in the data.

Chrom3D is based on Monte Carlo (MC) optimization with the goal of minimizing a loss-score function. The optimization process starts from random self-avoiding chromosome structures. Using the constraints described above imposed by TAD-TAD and TAD-LMNA interactions, iteration invokes one of five predefined local bead moves, affecting one or multiple beads while preserving bead chain connectivity (Fig. 1a; Additional file 1: Figure S3). This is in contrast to previous MC-based genome modeling where each bead is moved independently [18, 24, 31]. We model the genome at TAD (and sub-TAD) resolution from 13,878 beads, each spanning ~230 kb. In the simulations, TADs constrained by LADs are pushed toward the NP while Hi-C-constrained interacting TAD pairs are attracted to each other. The resulting Euclidean distances are assessed through a loss-score optimized until convergence is reached (Fig. 1b). The result of one simulation is a 3D modeled structure of the entire human genome where the concept of chromosome territories is respected (Fig. 1c; see also below for analysis of modeled chromosome territories). Of note, the lamin constraint is neutral with respect to the detection of chromosome territories in the modeled structures (Additional file 1: Figure S4a).

**Fig. 2:**
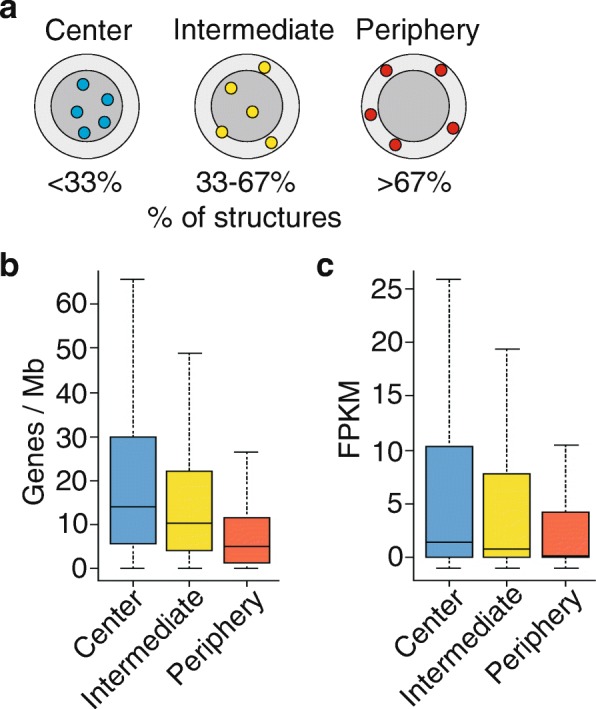
Characterization of TADs modeled at the NP and in the nucleus center. **a** Definition of periphery, center, and intermediate regions used to ascribe a radial position of TADs in Chrom3D structures. Volumes of the peripheral 1 μm thick “shell” (*light gray*) (NP) and of the nucleus “core” (*dark gray*) are equal, given a nucleus of 5 μm radius. A TAD is assigned to the NP if placed in the shell in > 67% of 400 structures, to an “intermediate” location if placed in the shell in 33–67% of the structures, and to the center if placed in the shell in < 33% of the structures. **b** Gene density and (**c**) gene expression level in TADs positioned at the periphery, center, or intermediate regions across 400 structures. FPKM values in (**c**) are from RNA-sequencing data downloaded from NCBI GEO accession number GSE33480

Since the optimization method is non-convex, a given simulation run may result in the representation of a structure from a local optimum in the loss-score function. Thus, to obtain a statistical estimate of the variability in the optimized structures, we generated 400 structures, each from 2 × 10^6^ iterations. We show that TADs associated with LMNA are mainly placed towards the NP whereas interacting TAD pairs without a LMNA-directed peripheral positional constraint are more evenly distributed in the nucleus (Fig. 1d, e). Interaction matrices reconstructed from the modeled structures show strong correlation with matrices generated from input Hi-C data for all chromosomes, providing validation to the structures (Additional file 1: Figure S5). We then defined the NP as a 1 μm thick outer “shell” partitioning the modeled nucleus into two compartments of equal volumes (Fig. 2a). As expected from microscopy observations, computation of chromatin (bead) density as a function of distance from the nucleus center shows that chromatin is not uniformly distributed in the modeled nuclei (Additional file 1: Figure S4b). Moreover, we find that across the 400 structures, gene density and expression level are lowest in TADs positioned at the NP (Fig. 2b, c). This is consistent with the gene-poor content and overall heterochromatic state of chromatin in this compartment [9]. Chrom3D therefore enables the reconstruction of 3D genome structures including a LMNA-directed constrained positioning of loci at the NP.

### Comparison of Chrom3D with IMP

We next compared Chrom3D with IMP, a popular framework for ensemble 3D genome modeling. To this end, we customized IMP to include LAD information as radial positional constraints. Simulation time is slightly faster with Chrom3D (Additional file 1: Figure S6a). Importantly, IMP tends to draw LMNA-containing TADs (beads) to the NP by stretching distances between consecutive beads, thereby violating chain continuity, especially for TADs associated with LMNA (Additional file 1: Figure S6b, c). We attributed this to IMP’s permutation strategy which involves randomly repositioning single beads, whereas by design Chrom3D always connects beads (Additional file 1: Figure S6b, red line). Moreover, using many beads, IMP generates large and intermingled chromosomes (Additional file 1: Figure S6d, e) that are less ellipsoidal and with greater variation in asphericity, beyond the 1–2 μm radius of chromosome territories estimated from microscopy studies [39] (Additional file 1: Figure S6f, g). This is likely due to initialization in an unconnected configuration. Thus, despite IMP’s suitability for 3D genome modeling of coarse-grained systems, Chrom3D more favorably models 3D genome structures with bead sizes at TAD and sub-TAD resolution from the constraints imposed in our system.

### Chrom3D is able to model local chromatin conformation

We assessed whether Chrom3D was scalable to restriction fragment-level size by modeling the ENCODE ENm008 region containing the α-globin locus, whose 3D conformation has been inferred from 5C data [18]. Clustering of 1000 Chrom3D-simulated conformations with no lamin constraint (see “Methods”) reveals greater structural variability in erythroleukemia K562 cells where the α-globin gene is expressed, than in lymphoblastoid GM12878 cells where it is repressed (Additional file 1: Figure S7a). Chrom3D structures also show compactness of the locus consistent with the previous structure and fluorescence in situ hybridization (FISH) data and with expression of the gene in these cell types [18] (Additional file 1: Figure S7b–d). We conclude that Chrom3D is also suited for structural chromatin modeling at the gene locus level.

### TADs associated with LMNA are more centrally placed than those associated with LMNB1

**Fig. 3:**
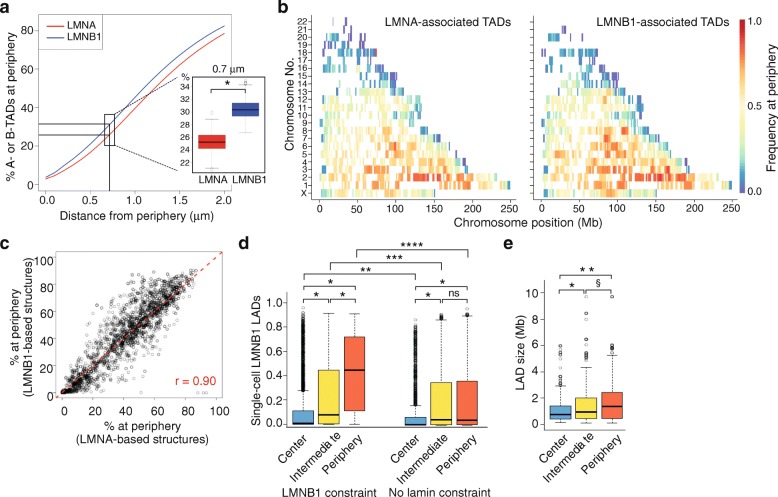
Chrom3D genome structures recapitulate features of genome organization estimated from single-cell analyses. **a** Percentage of LMNA-associated or LMNB1-associated TADs at a given distance from the NP across 400 structures; data are shown for TAD placement using LMNA or LMNB1 as radial constraint. *P* value for difference between the two curves is 9.53 × 10^–7^ (Wilcoxon rank sum test). Inset: percentages of LMNA-associated or LMNB1-associated TADs positioned at 0.7 μm from the nucleus edge shown for comparison with FISH data for LADs (see [41]); *P* < 2.2 × 10^–16^ (Mann–Whitney U test). **b** *Heatmaps* of radial stability of LMNA-associated or LMNB1-associated TADs in all chromosomes across 400 structures. “*Blue*” TADs are more stably placed in the nucleus center than “*red*” TADs, which are more stably placed at the NP. **c** Correlation analysis of lamin-associated TAD placement at the NP across 400 structures modeled with LMNA or LMNB1 constraints. **d** Comparison of TAD placement TADs modeled by Chrom3D with LMNB1 association frequency from the LMNB1 DamID in single cells [44]. *Left*, proportions of center, intermediate, and peripheral TADs in our structures associated with LMNB1 in the single-cell dataset; *P < 2.2 × 10^–16^ (Mann–Whitney U tests). *Right*, same as above, for TADs whose placement was modeled without lamin constraints; *P < 2.2 × 10^–16^; ^ns^ *P* = 0.755 (Mann–Whitney U tests). Comparisons between LMNB1 and no lamin constraint data: **P = 4.34 × 10^–5^, ***P = 6.20 × 10^–5^, ****P < 2.2 × 10^–16^, Mann–Whitney U tests. **e** Size distribution of LMNB1 LADs [44] in the structures; *P = 1.17 × 10^–3^; **P = 1.23 × 10^–6^; ^§^ *P* = 6.32 × 10^–3^ (Mann–Whitney U tests)

Association of TADs with the nuclear lamina and interactions between TADs reflect complementary but also potentially conflicting information on spatial positioning: two TADs may be predicted to interact, but peripheral assignment of only one (if associated with LMNA) may preclude them from being juxtaposed. Accordingly, we find that 40% of TADs associated with LMNA are placed in the peripheral 1 μm shell in our structures (Fig. 3a). This indicates that not all TADs associated with LMNA can be assigned to the NP in a given structure. We next modeled the radial placement of TADs in 400 new structures modeled using either LMNA or LMNB1 [7] as peripheral constraints. The results show that a LMNA constraint is less consistent with TAD placement at the NP than a LMNB1 constraint (Fig. 3a; *P* = 9.53 × 10^–7^; Wilcoxon signed-rank test), in line with a role of LMNA in chromatin organization also in the nuclear interior [6, 40].

Previous FISH analysis simultaneously probing 25 LMNB1 LADs in single HT1080 cells show that only 32% of LADs can be simultaneously detected at the NP (defined there as < 0.7 μm, or 8 pixels, from the nucleus edge) in a given nucleus [41]. This proportion is remarkably similar to that of TADs associated with LMNB1 localized within 0.7 μm of the nucleus edge across our 400 Chrom3D structures (30.5%; Fig. 3a, inset). It is also higher than that of LMNA-associated TADs modeled at the periphery (25%; *P* < 2.2 × 10^–16^; Mann–Whitney U test). This again indicates that not all LADs can be assumed to be found at the NP in individual nuclei in a cell population. This may be because some regions only transiently contact nuclear lamins at the NP and are therefore mainly detected in the nuclear interior.

### Assessment of chromatin stability at the nuclear periphery

Our previous results suggest that Chrom3D can recapitulate structures of genome conformation at the single-cell level. To further assess this contention, we examined the consistency of assignment of TADs at the NP across structures, with the rationale that this would reflect stably positioned TADs in this compartment across cells in a population. Chromosomal heatmaps of radial placement of TADs in structures modeled using LMNA or LMNB1 constraints reveal TADs with constitutive placement at the NP (<1 μm from the nucleus edge) or in the nucleus center and TADs with intermediate placement (Fig. 3b). As expected, the most stable peripheral TADs are located on the largest and most gene-poor chromosomes, while smaller gene-rich chromosomes harbor TADs more centrally placed (Fig. 3b). We find, however, no correlation between TAD size and peripheral stability of TADs across structures, indicating that the attribution of TADs from large chromosomes to the NP is not merely caused by TAD size (r = 0.16; Additional file 1: Figure S8). There is also concordance of radial positioning of TADs based on LMNB1 or LMNA constraints (Fig. 3b, c), consistent with the bulk of LMNA being enriched in the peripheral lamina. Moreover, subtelomeric regions appear overall more centrally placed than pericentromeric regions that are more stably ascribed to the NP (Fig. 3b), corroborating previous FISH data [42, 43]. The patterns and consistency of radial assignment of TADs across our ensemble of structures indicate that Chrom3D can capture principles of chromatin organization in single cells.

### Features of LADs estimated from the structures concur with lamin-genome contact patterns observed in single cells

We next compared our three radial placement categories (center, intermediate, periphery; see Fig. 2a) with single-cell NP-genome contact frequencies. These were defined by association of chromatin with the nuclear lamina observed in a previous single-cell LMNB1 DamID study in the near-haploid KBM7 cell line, the only cell type for which to our knowledge LADs have been mapped at the single-cell level [44]. Despite the difference in ploidy between HeLa and KBM7 cells, our structure ensembles reveal features of genome organization inferred from the single-cell observations. Indeed, TAD sequences assigned to the periphery in our structures show the highest peripheral contact frequency calculated from the single-cell LMNB1 DamID data and, conversely, the central category shows the lowest peripheral contact frequency in the single-cell dataset (Fig. 3d). Repeating this comparison excluding the lamin constraint in our modeling shows strongly reduced assignment of the regions to the periphery (Fig. 3d; *P* = 4.34 × 10^–5^ to *P* < 2.2 × 10^–6^; Mann–Whitney U tests). Moreover, focusing on LADs, our structures predict that larger LADs are more stably assigned at the periphery than smaller LADs (Fig. 3e; *P* = 1.23 × 10^–6^; Mann–Whitney U tests), again in agreement with the single-cell observations [44]. Our predictions of lower gene density and expression level in peripheral TADs (see Fig. 2b, c) are also supported by the single-cell data. We conclude that our ensemble of structures reflects the radial localization of LADs observed in single cells.

### FISH validates LADs modeled in the nuclear periphery and nuclear interior

**Fig. 4:**
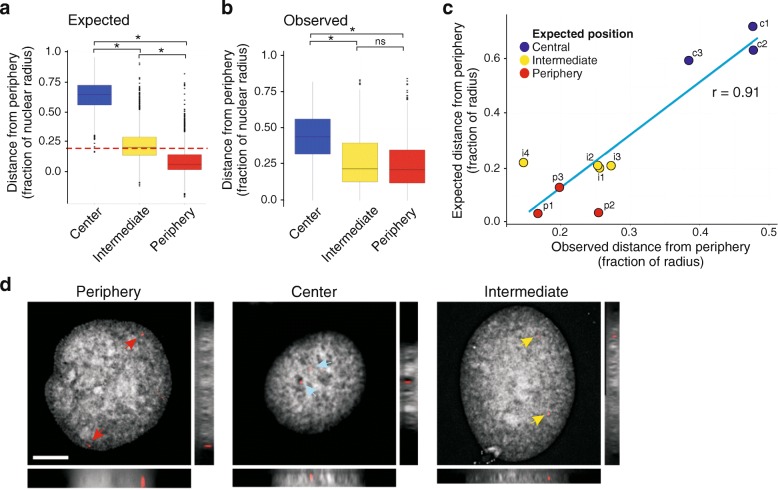
Validation of Chrom3D structures by FISH. **a** Expected radial distribution of ten FISH probes designed to the nucleus center (3 c probes), intermediate (4 i probes), and peripheral (3 p probes) areas, across 400 structures; *P < 2.2 × 10^–16^ (Mann–Whitney U tests). **b** Observed radial distribution of 1105 FISH signals for the ten probes analyzed in (**a**); *P < 10^–4^; ns, non-significant (Mann–Whitney U tests). Quantitative data for each individual probe are shown in Additional file 1: Figure S9b, c. **c** Correlation between observed and expected relative distance of FISH signals to the NP. **d** Examples of FISH images for each radial category (planar and orthogonal views); periphery, probe p1; center, probe c3; intermediate, probe i1. Bar, 5 μm

To validate the position of LADs predicted from the modeled structures, we carried out a FISH analysis. We analyzed 1105 FISH signals obtained from FISH probes designed to LMNA LADs placed at the NP, towards the nucleus center or in intermediate radial positions in the structures (Additional file 1: Table S1 and Figure S9a). The observed radial distribution of LADs from FISH analysis strongly concurs with predictions from the modeled structures (Fig. 4a–d; r = 0.91; Additional file 1: Figure S9b–d). To further appreciate the spatial coverage of individual chromosomes, four FISH probes were designed to chromosome 4 (Additional file 1: Figure S9e, f). Observed distributions of 453 FISH signals again agree with their predicted distribution (r = 0.97; Additional file 1: Figure S9g–i). These results validate the structures and indicate that LADs can be found in peripheral and central nuclear compartments. LAD distribution across structures also recapitulates their localization visualized in single cells.

### Chrom3D reveals laminopathy-specific LADs and differential gene regulation in the nucleus interior

Mutations in LMNA cause laminopathies which affect specific tissues [45] through still largely unknown mechanisms. The roles of LMNA on chromatin organization and mobility [46, 47] suggest that laminopathies may involve altered interactions of LMNA with chromatin in distinct nuclear compartments. This, however, has not been examined due to a lack of suitable tools. To gain 3D insight on chromatin changes that might be associated with LMNA mutations, we used Chrom3D to model the radial distribution of LADs associated with wt or mutated LMNA.

**Fig. 5:**
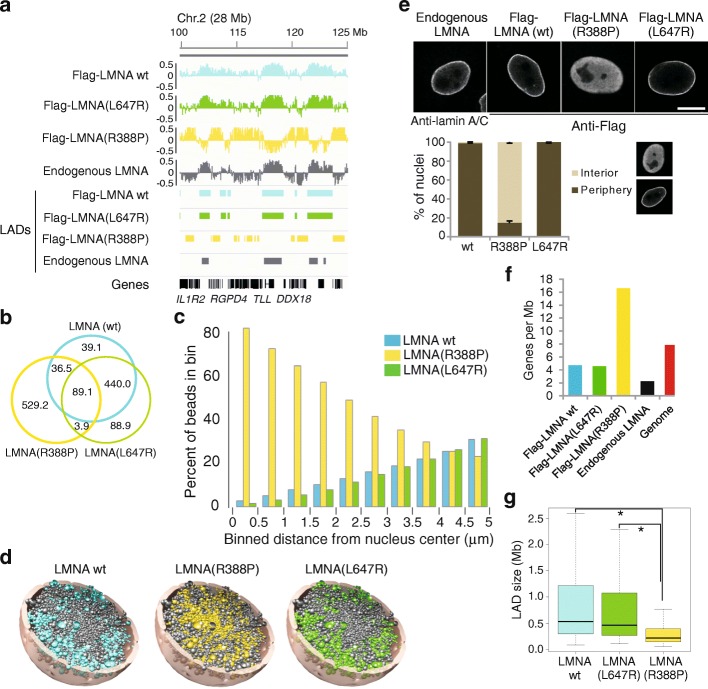
Differential radial positioning of TADs associated with peripheral and nucleoplasmic LMNA mutants. **a** ChIP-seq profiles (log(ChIP/input)) and corresponding LADs for flag-tagged LMNA wt, Flag-LMNA(L647R), Flag-LMNA(R388P), and endogenous LMNA in HeLa cells. **b** *Venn diagram analysis* of LAD overlaps (in Mb). **c** *Radial distribution* of TADs associated with Flag-LMNA wt, Flag-LMNA(L647R), and Flag-LMNA(R388P) across 400 structures modeled for each LMNA construct. **d** TADs associated with LMNA wt, LMNA R388P, or LMNA L647R (*colored beads*) superimposed onto all TADs (*gray beads*) in a modeled HeLa nucleus. **e** Immuno-localization of Flag-LMNA proteins and quantification of localization patterns (graph; > 300 nuclei per condition). **f** Gene density within indicated LADs and in the whole human genome. **g** LAD size distribution; *P < 2.2 × 10^–16^ (Mann–Whitney U tests)

First, we expressed in HeLa cells: (i) a flag-tagged version of a LMNA mutation, LMNA(R388P), causing congenital muscle dystrophy and lipodystrophy (Barateau et al., manuscript submitted); (ii) wt LMNA; and (iii) a LMNA(L647R) mutant causing a progeroid disorder [48] that localizes only at the NP by retention of its prelamin A-associated farnesyl moiety (Additional file 1: Figure S10a, b). We next mapped LADs associated with these LMNA proteins by ChIP-seq using anti-Flag antibodies. While LMNA wt and LMNA (L647R) LADs reveal strong overlap, there is little overlap between R388P LADs and wt or L647R LADs (Fig. 5a, b; Additional file 1: Figure S10b, c). Superimposition of wt and mutant LADs on 400 Chrom3D HeLa structures strikingly reveals that R388P LADs map more frequently to the nuclear center than wt or L647R LADs (Fig. 5c, d). Validating these predictions, immunofluorescence analysis shows that the LMNA(R388P) mutant is indeed distributed throughout the nucleoplasm, accounting for the intranuclear positioning of the majority of R388P LADs (Fig. 5e; Additional file 1: Figure S10d). Furthermore, R388P LADs are gene-rich (Fig. 5f) and narrower (Fig. 5g) than wt or L647R LADs (*P* < 2.2 × 10^–16^; Mann–Whitney U tests). These observations are again consistent with the radial placement of these LADs predicted by Chrom3D (Fig. 5c). These findings importantly indicate that Chrom3D can reveal radial positioning of loci without prior knowledge of their localization.

**Fig. 6:**
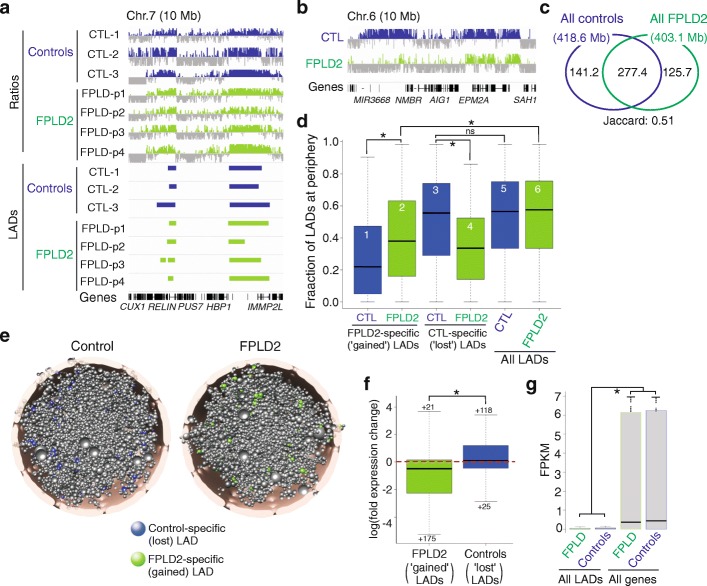
Chrom3D modeling of positioning of TADs containing laminopathy-associated LMNA LADs in FPLD2 patient cells reveals disease-specific LADs in the nuclear interior. **a** LMNA ChIP-seq profiles (shown as log(ChIP/input) ratios; scales: –3 to 3 centered on 0) and LADs in control and FPLD2 fibroblasts. **b** Example of differential LMNA enrichment patterns in control and FPLD2 fibroblasts. **c** Overlap of control and FPLD2 LADs (in Mb). Jaccard index of overlap is 0.51. Control-specific LADs (141.2 Mb) were determined by LAD regions found in at least one control cell type and not in any of the FPLD2 fibroblasts; FPLD2-specific LADs (125.7 Mb) were determined by LAD regions found in at least one FPLD2 fibroblast type and not in any of the controls. LADs were identified as described in “Methods.” **d** *Radial distribution* of all TADs containing LADs across 100 Chrom3D structures modeled from control and FPLD2 nuclei (bars 5.6) and of TADs containing FPLD2-specific LADs (bars 1, 2) and control-specific LADs (bars 3,4), both in control nuclei (*blue bars*) and FPLD2 nuclei (*green bars*); *P < 2.2 × 10^–16^ (Mann–Whitney U tests). **e** *Radial placement* of TADs containing control-specific LADs (*blue beads*) in a modeled control nucleus and of FPLD-specific LADs (*green beads*) in a modeled FPLD2 nucleus. **f** Fold-change expression level of genes within FPLD2-specific (“gained”) LADs in FPLD2 fibroblasts and within control-specific (“lost”) LADs in control fibroblasts; data expressed as log2(FPKM patients/FPKM controls); numbers are number of outliers; *P = 3.8 × 10^–9^ (Mann–Whitney U test). **g** Expression levels of all genes and of genes in LMNA LADs in FPLD2 and control fibroblasts; *P < 2.2 × 10^–16^ (Mann–Whitney U test)

Next, we determined whether Chrom3D could provide new insights into laminopathies by modeling the 3D genome in cells from patients harboring a LMNA mutation. We mapped by ChIP-seq using anti-lamin A/C antibodies, LMNA LADs in fibroblasts from four patients with familial partial lipodystrophy of Dunnigan type (FPLD2; OMIM#151160; patients “p1–p4”) bearing the same heterozygous LMNA p.R482W mutation [49] and in fibroblasts from three healthy control individuals. We examined the R482W LMNA mutant because the R > W substitution has been shown to impair DNA and nucleosome binding to LMNA in vitro [50, 51], and thus might affect LMNA association with chromatin in patient cells. Genome browser views of LMNA enrichment and detected LADs, and LAD overlap analyses, show that LMNA LADs in control and FPLD2 cells are overall conserved (Fig. 6a) but also show differences in genome coverage (Fig. 6b, c; Additional file 1: Figure S11a–c). Differential LMNA-chromatin association was corroborated by ChIP-quantitative polymerase chain reaction (qPCR) analysis of 16 genic and ten intergenic loci in fibroblasts from three patients and two controls (Additional file 1: Figure S11d, e).

Using Chrom3D, we generated 100 structures for each of the control and patient fibroblasts by integrating control and FPLD2 LMNA LAD datasets with TAD information from published Hi-C data for IMR90 human fibroblasts [38]. We find that strikingly, LADs specific to FPLD2 patients (LADs “gained” in FPLD2 fibroblasts) are more centrally located than all LADs in these cells (Fig. 6d, bars 2, 6; *P* < 2.2 × 10^–16^, Mann–Whitney U test); these domains are also centrally placed in control cells (Fig. 6d). In contrast, LADs unique to control fibroblasts (“lost” in patient cells) are found at the NP, to the same extent as all LADs in these cells (Fig. 6d; bars 3, 5; *P* = 0.35). Figure 6e shows examples of peripherally placed “lost” LADs in a modeled control nucleus and centrally placed “gained” LADs in an FPLD2 nucleus. We nevertheless note that a gain or loss of LADs is associated with partial recruitment of these regions towards, or away from, the NP, respectively (Fig. 6d, bars 1, 2; *P* < 2.2 × 10^–16^ and bars 3, 4; *P* < 2.2 × 10^–16^). These findings imply that LADs gained in patient cells are mainly restricted to the nuclear interior and are unexpectedly not fully repositioned to the NP. In addition, a gain or loss of LADs correlates with overall downregulation or upregulation, respectively, of gene expression within them (Fig. 6f; *P* < 2.2 × 10^–16^, Mann–Whitney U test), providing functional significance to the differential LMNA associations identified in FPLD2 patient cells. This is in a context of similar range of expression levels of all genes and of genes within LADs, both in patient and control cells (Fig. 6g).

Providing additional biological meaning to the modeled structures, Gene Ontology enrichment analysis highlights distinct functions of genes found in control-specific LADs (signaling and metabolic processes) and FPLD2-specific LADs (developmental processes; Additional file 1: Table S2). Interestingly, relevant for the metabolic phenotype of FPLD2 patients [49], the gained and lost LADs in patient fibroblasts contain genes implicated in white and brown adipocyte differentiation and metabolism (e.g. *PRDM16*, a master regulator of adipose tissue browning, *RARRES2*, *LGR4*, *BCATENIN*, *PLCB1*, *PTGS2*, *FABP4*, *RSPO3*, and *EIF2AK3*). Predictions emerging from this modeling therefore suggest that adipogenic and metabolic defects in FPLD2 patients with the LMNA p.R482W substitution might be associated with a deregulation of LADs in the nuclear interior and not exclusively at the nuclear envelope. This could speculatively involve differential binding of lamin A/C to promoters [52]. Our 3D genome modeling framework paves the way to more targeted investigations of disease mechanisms affecting genome architecture.

## Discussion

We present Chrom3D, a software for 3D genome modeling based on the inclusion of positional input constraints from chromosomal interactions (Hi-C data) and nuclear lamin-chromatin associations (lamin ChIP-seq data). Several key features of our modeled structures are shown: inclusion of radial positional constraints, scalability from a single locus to the whole human genome, and predictive value of radial placement of TADs. Chrom3D is versatile in that other positional constraints can be integrated. Finally, we show an application of Chrom3D to the study of disease mechanisms using FPLD2 patient-specific positional constraints imposed by a LMNA mutant displaying alterations in its association with the genome. Incorporation of radial positional constraints provides new insight into the placement of genomic regions in the 3D mammalian nucleus space with respect to the NP, which has not been possible from current genome modeling platforms. Our ensembles of structures reveal information on the cell-to-cell variation in genome structures likely to exist in a cell population. They recapitulate the peripheral positions of TADs in single cells and notably ascribe a subset of LMNA-associated TADs, and thereby LADs, in the nuclear interior without prior knowledge of such localization. Inference of an intranuclear localization of LMNA LADs concurs with the nuclear distribution of A-type lamins [40] and with their association with euchromatin, including active genes [6, 7, 53], which is enriched in the nuclear interior. Our structures therefore have predictive capacity. We exploit this property to infer the internal positioning of LADs associated with a pathological LMNA(R388P) mutation, after superimposition of these LADs onto structures. This concurs with the only information currently available on this lamin mutant, namely LAD data determined by ChIP-seq of an epitope-tagged version of this mutant, and its localization throughout the nucleoplasm visualized by immunostaining. Our findings illustrate the benefit of optimization-based 3D modeling to unveil the interplay between factors determining 3D genome structure.

The predictive capacity of our structures has important implications in understanding the relationship between genome structure and disease [54, 55]. Chrom3D structures enable a gain of spatial insight into disease-causing mechanisms, e.g. laminopathies as illustrated here. The structures reveal how alterations in LMNA-chromatin associations specific to FPLD2 patients with the LMNA(R482W) substitution predictively occur centrally in the nucleus and not necessarily at the NP as one might have expected. This opens the door to better targeted molecular investigations of the disease. Our modeling approach should not only be applicable to other laminopathies, but also potentially to diseases linked to dysfunction or mis-regulation in other nuclear components.

Challenges remain, however, before 3D genome modeling can be routinely applied in disease contexts. First, the genome must be modeled at appropriate spatial resolution to infer significant associations between genome structure and disease mechanisms; this may be required, for instance, to place selected genes and other genomic elements into correct regulatory neighborhoods. We have modeled the genome at TAD and sub-TAD resolution, providing high resolution structures of the diploid human genome. We do not imply that TADs exist as structural units at the single-cell level, but TADs reflect statistically enriched topological domains that prove to be relevant units for modeling. Second, the size and complexity of the human genome necessitate some level of coarse graining for any 3D modeling exercise. Thus, a tradeoff between resolution and throughput of structures is inevitable: we have focused here on an elevated number of beads in our structures and a smaller ensemble of structures. Nevertheless, we show how critical insights into cell-to-cell variability of genome structures can be gained from radial positioning constraints.

## Conclusions

Chrom3D is a genome 3D modeling platform integrating Hi-C data together with positional constraints from the association of loci with intranuclear anchors such as nuclear lamins. While pairwise domain interactions are important to enforce contacts between distal genomic regions, radial positioning provides key information on the spatial organization of genomic domains. Incorporation of radial positioning constraints in 3D genome structures enables the study of spatial gene regulation in disease, for example so-called nuclear envelopathies, caused by mutations in nuclear envelope proteins. Extending positional information to other chromatin anchor points in the nucleus should expectedly enhance applications of 3D genome structures to the study of disease mechanisms.

## Methods

### Cells

HeLa cells (American Type Culture Collection; CCL-2) were cultured in MEM medium containing Glutamax (Gibco), 1% non-essential amino acids and 10% fetal calf serum. Cells were transfected using XtremeGENE 9 (Roche) using a 3:1 ratio (μL:μg) of X-tremeGENE 9 DNA Transfection Reagent and DNA. Primary skin fibroblast cultures were established from healthy volunteers aged 20 years and 33 years (CTL-1, CTL-3) and from four patients with familial partial lipodystrophy of Dunnigan type (FPLD2) due to a LMNA p.R482W heterozygous mutation (female, age 43 years (“FPLD-p1” patient), female, age 37 years (FPLD-p2), female, age 14 years (FPLD-p3), male, age 43 years (FPLD-p4) [56]. These studies were approved by the Institutional Review Board of Hôpital Saint Antoine (Paris, France). Normal skin fibroblasts were also purchased from Lonza (“CTL-2”). Fibroblasts were cultured in DMEM/F12/10% fetal calf serum, 10 ng/mL epidermal growth factor, 24 ng/mL basic fibroblast growth factor, and 1% Penicillin-Streptomycin. Cultures were at passage 5–7 when used.

### Plasmids

pCMV-Flag-preLA-WT and pCMV-Flag-preLA-L647R vectors were generated by *LMNA* amplification of pSVK3-Flag-preLA-WT and pSVK3-preLA-L647R [50], with the 5′ CCGGATCCTATGGAGACCCCGTCCCAGCGG-3′ and 5′ GCGAATTCTTACATGATGCTGCAGTTCTG-3′ primers and insertion of the PCR product into pCMV-Flag at *BamH*1 and *EcoR*I sites. pCMV-Flag-preLA-R388P was constructed from pCMV-Flag-preLA-WT using the QuikChange Lightning Site-Directed Mutagenesis Kit (Agilent Technologies). pCMV-Flag-preLA-wt was amplified by PCR using 5′-GAGGAGAGGCTACCACTGTCCCCCAGC-3′ and 5′-GCTGGGGGACAGTGGTAGCCTCTCCTC-3′ primers, products digested by *Dpn*I and XL10-Gold® ultracompetent cells were transformed. pEGFP-preLA-R388P was constructed by *LMNA* amplification of pCMV-Flag-preLA-R388P with the 5′ GCCCTAGGTGAGGCCAAGAAGCAACTT 3′ and 5′ GCCCATGGACTGGTCCTCATTGGACTTGT 3′ primers and insertion of the PCR product into pEGFP-preLA-wt at *EcoN*I and *PflM*I sites.

### Immunofluorescence

Cells grown on coverslips were fixed 24 h after transfection with 3% paraformaldehyde, permeabilized in PBS/0.5% Triton X-100, and incubated in PBS/0.1% Triton X-100/2% BSA for 25 min. Cells were incubated for 30 min each with primary and secondary antibodies in PBS/0.1% Triton X-100/1% BSA. Antibodies were anti-Flag (1:200; Sigma), anti-lamin A/C [50] (1:400), and anti-rabbit Alex Fluor® 594 (1:200; Jackson ImmunoResearch). DNA was stained with Hoechst 33258. Coverslips were mounted with Mowiol and examined on a LSM 700 confocal microscope (Zeiss) at the Imaging Facility of the Functional and Adaptive Biology Unit of University Paris Diderot/CNRS.

### Immunoblotting

Proteins were separated by SDS-PAGE and transferred onto nitrocellulose. Membranes were incubated with anti-lamin A/C [50] or anti-GAPDH antibodies (1:15,000; Sigma) and with horseradish peroxidase-conjugated antibodies (1:20,000; Promega). Signals were detected by enhanced chemiluminescence.

### RNA-sequencing

Total RNA was isolated from control and FPLD2 fibroblasts using the Ambion TRIzol® Reagent RNA extraction kit (Life Technologies) [57]. Libraries were sequenced on an Illumina HiSeq2500. RNA-sequencing (RNA-seq) reads were processed using Tuxedo [58]. TopHat [59] was used to align reads to hg19 applying the Bowtie 2 [60] preset “very sensitive.” Gene ontology analysis was done with topGO in R [61].

### ChIP of LMNA and LAD identification

ChIP of Flag-LMNA proteins in HeLa cells was done using anti-Flag antibodies (20 μg/10^7^ cells) as described [57]. ChIP of LMNA from fibroblasts was done using anti-lamin A/C antibodies [57]. Illumina libraries were sequenced on a HiSeq2500. DNA was also used as template for qPCR (Additional file 1: Table S3), with 95 °C for 3 min and 40 cycles of 95 °C for 30 s, 60 °C for 30 s, and 72 °C for 30 s. Sequence reads were aligned to hg19 genome using Bowtie2 with default parameters and option -best enabled. LADs were called using Enriched Domain Detector [57] using a 1-kb bin size and default parameters. Browser files were generated from the ratio of ChIP/input for each 1-kb bin with input normalized to ratio of [total ChIP reads/total input reads]. Scripts were written in Perl [62] or R [61].

### TAD and bead definition

Genomic positions of TADs were based on contact domains identified from Hi-C data [38] accessed under GEO GSE63525. Overlapping TADs were merged into single domains and regions not covered by a TAD were assigned a bead of size proportional to the corresponding genomic region. Bead sizes were scaled so that total bead volume constituted 15% of the volume of a 10-μm diameter modeled nuclei [63], using a previous scaling function [24].

### Assigning lamin information to TADs

TADs that overlap, fully or partially, with a called peak from lamin ChIP-seq data (i.e. a LAD) [57] were designated as LMNA-associated or LMNB1-associated TADs and were constrained towards the NP. This resulted in 1718 LMNA-associated TADs (see Additional file 1: Figure S1 and S2, blue segments) and 2770 LMNB1-associated TADs.

### Inference of significant interactions from Hi-C data

Interactions between beads were defined from high-resolution Hi-C data for HeLa cells [38] accessed under dbGap number phs000640. To infer statistically significant interactions, we adapted the ChiaSig method designed for ChlA-PET [64] to Hi-C. To this end, we estimated the dependency between linear genomic distance and contact frequencies using 1-Mb bins. Refinement of genomic distance–contact frequency relationship was not necessary because most pairwise combinations of bins reflect background looping information. To estimate background distribution for inter-chromosomal interactions, we used the average number of inter-chromosomal interactions between all pairs of bins between chromosomes. ChiaSig calculates a *P* value based on the probability of observing a given number of contacts conditional on the total number of contacts for both regions involved, as well as the total number of contacts, using a non-central hypergeometric distribution. This adjusts for the propensity of different regions to be involved in contacts, including technical bias (GC-content, accessibility). Intra-chromosomal interactions were selected with FDR 0.01% [21]. For inter-chromosomal interactions, we also required that interactions be significant in HeLa cells and in > 4 of the seven cell lines analyzed previously (GM12878, HMEC, HUVEC, IMR90, K562, KBM7, NHEK) [38].

This resulted in 3824 significant interactions (3657 intra-chromosomal and 167 inter-chromosomal) for HeLa (see Additional file 1: Figure S2, red segments). For IMR90, we obtained 2349 significant interactions (1558 intra-chromosomal and 791 inter-chromosomal). These interactions were associated with TADs by mapping the mid-point of each Hi-C bin to the corresponding TAD. This resulted in 2586 beads for HeLa cells and 1744 beads for IMR90 (each × 2 to account for a diploid genome) with at least one interaction.

### Peripheral, central, and intermediate assignment of TADs in the modeled structures

To examine genomic properties of TADs as a function of radial position in the modeled nuclei, we divided the nucleus into a peripheral “shell” 1.03-μm thick and a central compartment, each making up 50% of the total nucleus volume. A TAD was assigned to:

- the NP if placed in the shell in > 67% of 400 structures;
- an “intermediate” location if placed in the shell in 33–67% of the 400 structures;
- the nucleus center if placed in the shell in < 33% of the 400 structures.

### Chromatin modeling framework

We developed a software suite for MC optimization and modeling of chromatin 3D structure using C++. Simulations were done using this software, except for when IMP was used for comparison. The concept is to enable incorporation of constraints and enable MC optimization by invoking local perturbations on chromosome regions. These chromatin “moves” have the favorable property that they alter only a small part of chromatin structure in each iteration, while maintaining connectivity of the chromatin chain. This is in contrast to previous MC-based methods [28] where each bead is moved independently. During simulation, moves are selected randomly according to weights specified by the user. In all simulations carried out here, these weights were set equal, such that each move has the same chance of being selected in each iteration. For a given structure, we defined a loss-score (L) as the sum of the individual loss-scores of each constraint [18] (Equation 1):

$$ L={\displaystyle \sum_{i,j}{k}_{ij}}{\left(\left\Vert {b}_i-{b}_j\right\Vert -{d}_{ij}\right)}^2, $$

where the sum runs over all bead positions where a constraint has been defined and *d*_*ij*_ is the target Euclidean distance of the given pair of beads *i* and *j.* Beads to be associated with the NP are optimized according to the distance from a “dummy bead” assigned in the nucleus center (the origo). The dummy bead has a radius of 0 and is in all instances (except for loss-score calculations) not considered as part of the modeled structure. Each constraint can be weighted by a factor *k*_*ij*_ *,* to allow for selected constraints to be prioritized in the MC optimization. Target distance of all bead pairs constrained by a Hi-C interaction between them was set to the sum of the radii of the two beads, effectively minimizing the distance between them without bead overlap. For beads constrained by LADs, target distance (from the nucleus center) was set to the difference between the nucleus radius and the bead radius, allowing lamin-constrained beads to move to the nuclear “wall.” All non-lamin beads were pulled towards the center by minimizing their distance to the nucleus center (“dummy bead”). At the start of each simulation, we initialize the modeling based on self-avoiding random walk structures sampled such that none of the chromosomes clash or overlap. We perturb the structure using the “moves” and minimize the loss-score using simulated annealing. A move was accepted based on resulting Euclidian distances between interacting TADs or between TADs and the NP, according to the Metropolis–Hastings algorithm with simulated annealing. Moves causing a clash between beads were discarded.

Chrom3D software and documentation can be freely accessed at: https://github.com/CollasLab/Chrom3D.

The version of the source code used in the manuscript is available at: https://doi.org/10.5281/zenodo.168212

### Comparisons of Chrom3D with IMP

We developed a modeling procedure based on IMP using the same set of constraints and number of beads as for Chrom3D. We initialized TAD beads as particles and added “ExcludedVolumeRestraint” to disable bead–bead clashes. For each consecutive pair of beads on each chromosome, we added harmonic springs with a spring distance equal to the two radii of the beads. Interactions between non-consecutive beads (based on Hi-C) were modeled using harmonic springs with a distance corresponding to the sum of the radii of the bead pair, similarly to Chrom3D. Beads with lamin constraints were pushed to the NP using the “HarmonicLowerBound” and a “dummy bead” placed in the nucleus center. Spring distance from the lamin-bead and the dummy bead was set to nucleus radius (5 μm) minus bead radius. To run MC optimization, we used “MonteCarloWithLocalOptimization” with fie local steps and a total of 500 iterations, which was sufficient to reach convergence. Ten independent simulations were done for comparison with Chrom3D structures.

To compare chromosome territories, we used the radius of gyration, calculated as the root mean square distance of the beads on each chromosome from their common center of mass (using bead volume to represent the mass). To estimate individual chromosome deviations from a spherical shape, we used the asphericity measure based on the eigenvalues of the gyration tensor [65].

### Modeling of the α-globin gene locus

The ENm008 ENCODE region containing the α-globin locus was modeled using Chrom3D based on published 5C chromosome conformation capture data [18]. For each restriction fragment, we created beads (n = 70) of diameter corresponding to the genomic length of the fragment multiplied by 0.005 [18]. Preprocessing and distance conversion rules for 5C data were as described [18]. In contrast to the input file used for earlier 3D reconstruction [18], we did not include distance constraints between neighboring beads since our modeling framework represents each chromosome as a chain of connected beads. Thus, distance constraints included non-interaction constraints (two beads should not get closer than a given distance) and interaction constraints (two beads should not get further from each other than a given distance). For all bead pairs with zero contacts detected in the 5C contact matrix, we used non-interaction constraints. Thresholds for non-interaction and interaction distances were cell type-specific (K562 and GM12878 cells) [18]. We ran 1000 simulations for each cell line using 40,000 iterations and a cooling rate of 0.000125, excluding whole chromosome “Translation” and “Rotation” moves. Final structures were aligned using Procrustes analysis (procrustes method in the vegan R package; default parameters with no scaling) and clustered using agglomerative hierarchical clustering (agnes method in the cluster R package; metric = “manhattan”). We extracted the cluster containing highest proportion of simulated structures for each dataset and plotted these on top of each other with high transparency so that the most common positions for each bead are the most visible in the plot. Coloring scheme was approximated to the scheme used in Table 1 in [18]. Bead sizes (in base-pairs) were: min 2; median 5151; mean 7043; max 29,050; bead radii in nm: min 0.01, median 25.76, mean, 35.21, and max 145.20. For distance calculations for comparison with FISH probes, we used beads 14 and 58 to calculate their distances across all structures for each cell line.

### FISH probe design

The model nucleus was divided into two compartments at *r*_*half_v*_ = 3.97 μm distance from nuclear center (considering a 5 μm radius), each compartment being of equal volume. This provided two regions for bead placement: beads with centers located < *r*_*half_v*_ from the nucleus center were classified as central, and peripheral otherwise. Proportions of each bead placed in peripheral or central region across 400 structures were used to identify the most stable beads in the periphery or center. Beads were further filtered to select beads associated with LMNA [7] (GEO GSE57149; track GSM1376181). We also designed probes to beads that were neither stable in the periphery nor center (“intermediate” area). Additionally, due to the variable copy number of genomic segments in HeLa cells, probes were designed to avoid areas with high copy number variations. Positions of FISH probes are shown in Additional file 1: Figure S4a and FISH probe information is shown in Additional file 1: Table S1.

### FISH procedure and signal detection

Cells were incubated in hypotonic buffer (0.25% KCl, 0.5% tri-sodium citrate) for 10 min and fixed in ice-cold methanol:acetic acid (3:1). Cells were dropped on slides. BAC FISH probes (BacPac Resource Center) (Additional file 1: Table S1) were labeled using a Nick Translation Kit and Biotin-16-dUTP (Roche). Per slide, a 200–300 ng labeled probe was mixed with 8 μg human Cot-1 DNA and 30 μg salmon sperm DNA (Invitrogen) and precipitated. A DNA pellet was dissolved in 11 μL hybridization mix (50% deionized formamide (Ambion), 2× SSC, 1% Tween 20, 10% dextran sulphate) at 42 °C for 20 min and pre-annealed for 1 h at 37 °C. Slides were RNase-treated and washed twice in 2× SSC, dehydrated in 70%, 90%, and 100% ethanol, and air-dried. Slides were denatured for 1 min 20 s in 70 °C 70% deionized formamide/2× SSC, pH 7.5, dehydrated in ice-cold 70%, 90%, and 100% ethanol, and air-dried. Probes were denatured for 5 min at 70 °C and pre-annealed for 15 min at 37 °C. Ten microliters of probe were applied onto coverslips (22 × 22 mm) which were then mounted on a slide. Slides were hybridized overnight at 37 °C. Slides were washed in 2× SSC (45 °C 2 min then 3× 5 min) and in 0.1× SSC (60 °C for 4× 4 min). Slides were blocked in 5% skim milk in 4× SSC for 15 min at 37 °C and incubated at 37 °C for 30–60 min with Avidin Alexa Fluor 488 conjugate (Invitrogen) (1.7 μg/mL in blocking buffer). Slides were washed in 4× SSC/0.1% Tween 20 for 3× 5 min and incubated with Biotinylated Anti-Avidin D conjugate (goat; 1.0 μg/mL in blocking buffer) (Vector) for 30 min at 37 °C. Slides were washed and incubated with Avidin Alexa Fluor 488 conjugate as above. Slides were mounted with 0.2 μg/mL DAPI in Dako Fluorescent Mounting Medium.

A total of 484 FISH images were analyzed using FISHfinder [66] to detect probes and calculate their position relative to the nucleus edge (n = 1105 FISH signals). Images were taken in DeltaVision image stack format (.dv). Significance of FISH signal localization in central, intermediate, and peripheral regions was tested by Mann–Whitney–Wilcoxon tests.

### Data viewing

Browser views of ChIP-seq data are shown using Integrated Genomics Viewer [67]. Genes are from Illumina iGenomes gene annotation with UCSC source for hg19.

## Additional file

Additional file 1: Figure S1. Source data for Chrom3D modeling. **Figure S2.** Hi-C and LMNA constraints integrated in Chrom3D. **Figure S3.** Chromosome moves introduced in the Chrom3D modeling framework. **Figure S4.** Structure characteristics. **Figure S5.** Correlation analysis between interaction frequency matrices reconstructed from 400 modeled structures and matrices from input Hi-C data. **Figure S6.** Chrom3D and IMP comparison. **Figure S7.** Chrom3D modeling of the ENm008 ENCODE region containing the α-globin locus. **Figure S8.** Stability of TADs at the nuclear periphery as a function of TAD size, across 400 structures. **Figure S9.** FISH validation of radial TAD placement predicted by Chrom3D. **Figure S10.** Expression of Flag-LMNA wt, Flag-LMNA(R388P), and Flag-LMNA(L647R) in HeLa cells. **Figure S11.** LMNA LADs in FPLD patient and control fibroblasts. **Table S1.** FISH probe information. **Table S2.** Top 10 enriched GO terms for genes in LADs specific to control or FPLD patient fibroblasts. **Table S3.** ChIP-qPCR primers and genomic location of amplicons. (PDF 2914 kb)

## Abbreviations

ChIP: chromatin immunoprecipitation
FISH: fluorescence in situ hybridization
LAD: lamin-associated domain
LMNA: lamin A
LMNB1: lamin B1
NP: nuclear periphery
TAD: topologically associated domain
