# Supplementary material for: Chrom3D: three-dimensional genome modeling from Hi-C and nuclear lamin-genome contacts
Source: Genome Biol. 2017 Jan 30;18:21. doi: 10.1186/s13059-016-1146-2 (PMC5278575; doi:10.1186/s13059-016-1146-2)
Supplement: Additional file 1: Figure S1. — Source data for Chrom3D modeling. Figure S2. Hi-C and LMNA constraints integrated in Chrom3D. Figure S3. Chromosome moves introduced in the Chrom3D modeling framework. Figure S4. Structure characteristics. Figure S5. Correlation analysis between interaction frequency matrices reconstructed from 400 modeled structures and matrices from input Hi-C data. Figure S6. Chrom3D and IMP comparison. Figure S7. Chrom3D modeling of the ENm008 ENCODE region containing the α-globin locus. Figure S8. Stability of TADs at the nuclear periphery as a function of TAD size, across 400 structures. Figure S9. FISH validation of radial TAD placement predicted by Chrom3D. Figure S10. Expression of Flag-LMNA wt, Flag-LMNA(R388P), and Flag-LMNA(L647R) in HeLa cells. Figure S11. LMNA LADs in FPLD patient and control fibroblasts. Table S1. FISH probe information. Table S2. Top 10 enriched GO terms for genes in LADs specific to control or FPLD patient fibroblasts. Table S3. ChIP-qPCR primers and genomic location of amplicons. (PDF 2914 kb) [file 13059_2016_1146_MOESM1_ESM.pdf]

Additional file 1

Table S1 FISH probe information.

ProbeID	Placement	CloneID	Chr_FISH	Start_FISH	End_FISH	Chr_BEAD	Start_BEAD	End_BEAD
c1	Center	RP11-75H6	chr19	951641	1144485	chr19	0	1620000
c2	Center	RP11-91N21	chr21	15964909	16121996	chr21	15810000	16150000
c3	Center	RP11-516D7	chr4	189080551	189262582	chr4	189080000	190540000
i1	Intermediate	RP11-88D10	chr4	107517202	107724374	chr4	106600000	108670000
i2	Intermediate	RP11-59F18	chr6	112896841	113061362	chr6	112660000	114290000
i3	Intermediate	RP11-6F19	chr4	166935539	166958075	chr4	166770000	167010000
i4	Intermediate	RP11-81F21	chr2	205370883	205533061	chr2	204320000	206540000
p1	Periphery	RP11-337B18	chr2	163702326	163901711	chr2	163190000	163990000
p2	Periphery	RP11-79L13	chr2	163002269	163175338	chr2	163010000	163170000
p3	Periphery	RP11-96A1	chr4	120850223	121025851	chr4	120720000	121000000

Predicted locus localization across 400 genome structures:

Center
Intermediate
Periphery

Table S2 Top 10 enriched GO terms for genes in LADs specific to control or FPLD2 patient fibroblasts.

GO ID	Description	No. genes	Total No. genes in term	P-value
LADs gained in FPLD2 patient fibroblasts				
GO:0007399	nervous system development	155	2195	3.9e-07
GO:0010578	regulation of adenylate cyclase activity	8	18	8.0e-07
GO:0007626	locomotory behavior	27	205	2.1e-06
GO:0007423	sensory organ development	50	520	2.6e-06
GO:0050877	neurological system process	100	1316	3.0e-06
GO:0032501	multicellular organismal process	394	6936	9.2e-06
GO:0007610	behavior	60	701	1.1e-05
GO:0003008	system process	134	1955	1.3e-05
GO:0007187	G-protein coupled receptor signaling	22	179	5.2e-05
GO:0030182	neuron differentiation	94	1312	6.2e-05
LADs lost in FPLD2 patient fibroblasts				
GO:0009593	detection of chemical stimulus	532	60	4.4e-11
GO:0033139	regulation of peptidyl-serine phosph.	19	10	2.0e-09
GO:0002323	natural killer cell activation	30	12	2.5e-09
GO:0050909	sensory perception of taste	60	16	5.5e-09
GO:0007600	sensory perception	992	84	1.2e-08
GO:0050877	neurological system process	1316	102	3.0e-08
GO:0042501	serine phosphorylation of STAT protein	24	10	3.5e-08
GO:0007186	G-protein coupled receptor signaling	1291	100	4.3e-08
GO:0030101	natural killer cell activation	75	15	9.8e-07
GO:0030183	B cell differentiation	110	18	1.8e-06

Table S3 ChIP-qPCR primers and genomic location of amplicons.

Amplicon	Chromosome	Forward primer (F)<br>Reverse primer (R)	Genomic position (nt)
Chr3r1	Chr 3	F: CCCTCAGCATAGCGGTGTAG<br>R: AGAACTCAGTGAGGGCTTGC	38868181-38873708
Chr7r1	Chr 11	F: AGCTGTCTTCTGCCAGCAAT<br>R: ATGCCTTTGAGAGGCTGACC	141316843-141322985
Chr8r1	Chr 8	F: TGCTAGCTGACAGGTGATGC<br>R: GTTGTTCGTGCTAACGGCTG	6834856-6836060
Chr9r1	Chr 9	F: GAAGCCCCTTGGGTACCTTC<br>R: AGCCAGTGTCTGCTGGATTC	110660385-110663316
Chr11r1	Chr 11	F: CCCCCTCTGGTCTACAGGAT<br>R: GGAGTCTGGCAGGTAAACCA	59388905-59390474
Chr12r1	Chr 12	F: CCCTTCAAGGTGATGGGGTT<br>R: AGTGCAAGAACTTTCGGGCT	7833314-7833620
Chr12r2	Chr 12	F: CTCCCAGGCATGTCTCACTT<br>R: GAATGCTTTGCAGTCACCCC	129887824-129888838
Chr13r1	Chr 13	F: ACCTGCTGCTATGGTGCATT<br>R: GGCTGAAGTCTGCTGTGTCT	27414218-27417293
Chr14r1	Chr 14	F: AGCTAACAGGACAGAGTGCT<br>R: TGTGCATGGGAAGGTCTTGG	104288010-104288880
Chr21r1	Chr 21	F: GGTCCCCAACAGCCTTACAA<br>R: CCTGCACGAATCCTGACCTT	41458227- 41459362
Chr22r1	Chr 22	F: CTGGACCAGGGGAAACTGAC<br>R: GATTTTCAGCCACCCCCTGA	37524006-37524443
TAS2R38	Chr 7	F: CACTCAGGAACAGCAGTCCA<br>R: CTGACCAATGCCTTCGTTTT	
TCN1	Chr 11	F: ACTTGGTAGGGATGCTGTGC<br>R: AAAAGGGTGGCAGGAGATGG	
DNAL4	Chr 22	F: CTGGCTCTTTCCAAGGAGGG<br>R: TACGAAGCCACCCACAGTTC	
NANOG	Chr 12	F: AAGTCCTGGGAAGCGTTGAC<br>R: GCAGCTACCAGTCAGAAGCA	
SCN10A	Chr 3	F: TGTCACCAGTCCTTCCATGC<br>R: GGAAGAGGGTGAGGGAAAGC	
ACTL7A	Chr 9	F: GGATCAGGTTCAGGGTGTGG<br>R: GGACCTAGCGTGCAACTCTT	
KRT23	Chr 17	F: GACTGCCCTGGATGGTTTTA<br>R: ACAACAACAGTGTGCGAAGG	
RPA4	Chr X	F: TAGGGTGGGCTGAAAAACAG<br>R: CCGGATTGCTGGTTAAAGA	
BACE2	Chr 21	F: CCCAGCACCATAGGCTTGAA<br>R: GGATGGCAGAGCTGTCTTGT	
DEFA3	Chr 8	F: CCCGGGCATGGTCTAGAATC<br>R: AGCCCTTCACATCCATTGGG	
ATP5EP2	Chr 13	F: GTCAAACTGCACAGGGCTTG<br>R: CAGCCTGGTTCTGATGGTGT	

GYS2	Chr 21	F: CACAGGCTACAGGAGCACAA<br>R: AAGTGATGCCACAAGTGCCT
AJAP1	Chr 1	F: TTACCCTGGCAACACCATCC<br>R: TTGCGAAAGGCTCGAGAGAG
STX2	Chr 12	F: CCAAGCACCCAGCACTCTAA<br>R: CAAAGGACTGTGCACCAAGC
LDHB	Chr 12	F: TTGGAAATCCGAGTACCGCC<br>R: AAGCCTCTGGAAGGAGACCT
HOXB9	Chr 17	F: TCTCCTGTGGATTGCTGCTG<br>R: GCAGGCCCTCATTTCCTCTT

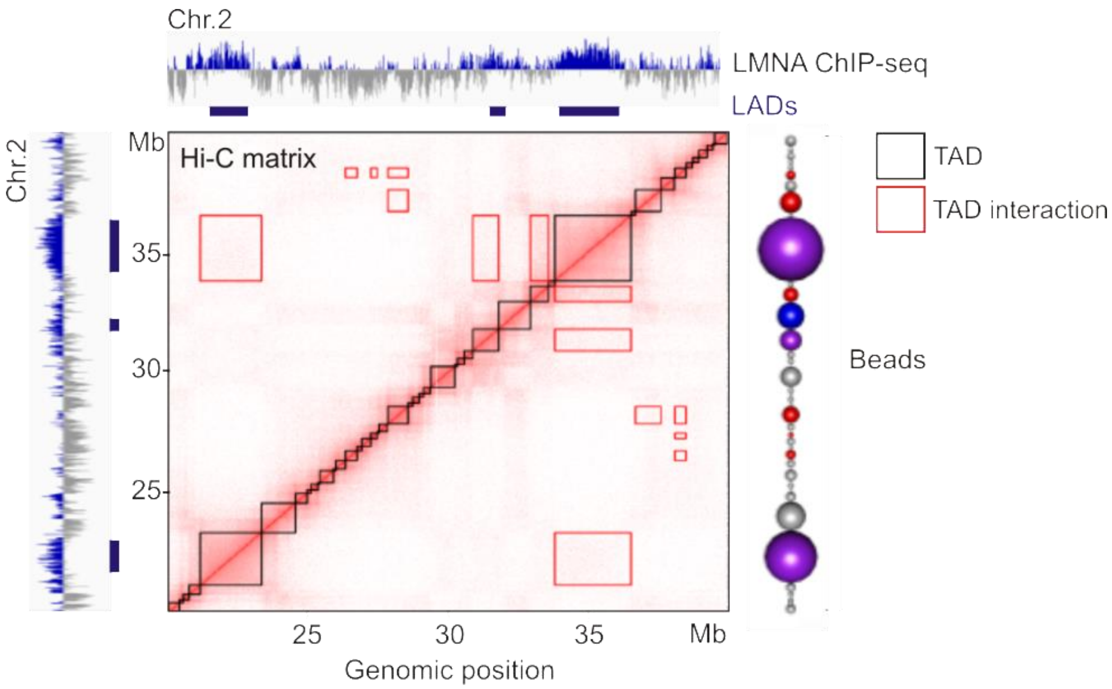

Fig. S1 Source data for Chrom3D modeling. Chrom3D is based on the integration of significant interactions between TAD pairs determined from Hi-C data (red boxes in the Hi-C matrix) and of interactions of TADs with the nuclear periphery. The latter is determined from the mapping of LMNA LADs from ChIP-seq data (enrichment ratios, and LADs shown in blue) and by ascribing a LAD to a TAD. This is shown here for a 20 Mb region of human chromosome 2. Each chromosome is modeled as a beads-on-a-string structure, where each bead corresponds to a TAD (black squares). The interaction constraints are imposed on the beads with significant interactions inferred from Hi-C data (red squares); here illustrated as red beads. TADs with only LAD constraints are shown as blue beads, while TADs with both LAD and significant Hi-C constraint are represented as purple beads. The TADs without any constraints are depicted as gray beads. See also Figure 1a for a schematic representation of Chrom3D principles.

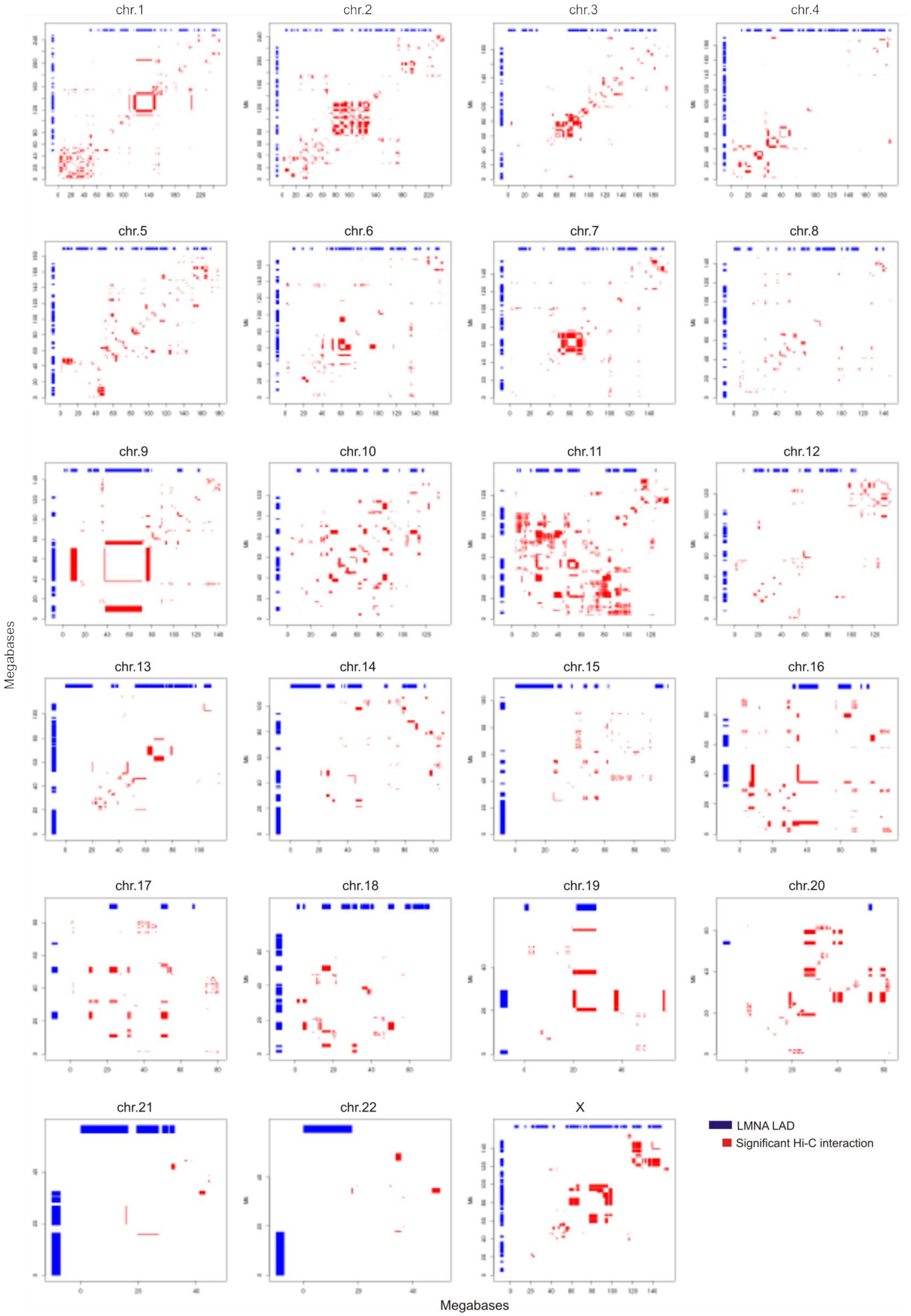
chr.1
chr.2
chr.3
chr.4
chr.5
chr.6
chr.7
chr.8
chr.9
chr.10
chr.11
chr.12
chr.13
chr.14
chr.15
chr.16
chr.17
chr.18
chr.19
chr.20
chr.21
chr.22
X
LMNA LAD
Significant Hi-C interaction
Megabases
Megabases

Fig. S2 Hi-C and LMNA-LAD constraints integrated in Chrom3D. LMNA-LADs mapped from LMNA ChIP-seq data in HeLa cells (blue bars) and significant interactions determined from high-resolution Hi-C data also in HeLa cells (red) are shown for all chromosomes.

Move	Description	Affected chromosome domain	Illustration
Crankshaft	Rotation of a central part of a chromosome (one or more beads) without perturbing coordinates of the rest of the chromosome	Part of chromosome	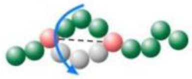
ArmRotate	Rotation of part of one of the chromosome arms without perturbing coordinates of the rest of the chromosome	Part of chromosome	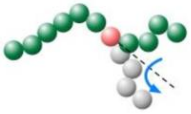
ArmWiggle	A random-walk re-sampling of one part of a chromosome arm, without perturbing coordinates of the rest of the chromosome	Part of chromosome	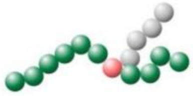
Translation	Shift in the position of all beads of a given chromosome without perturbing any other chromosome	Whole chromosome	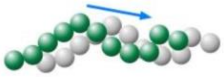
Rotation	Rotation of an entire chromosome along an axis defined by two randomly selected beads along the chromosome without perturbing any other chromosome	Whole chromosome	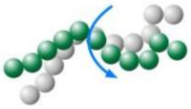

Fig. S3 Chromosome moves introduced in the Chrom3D modeling framework.

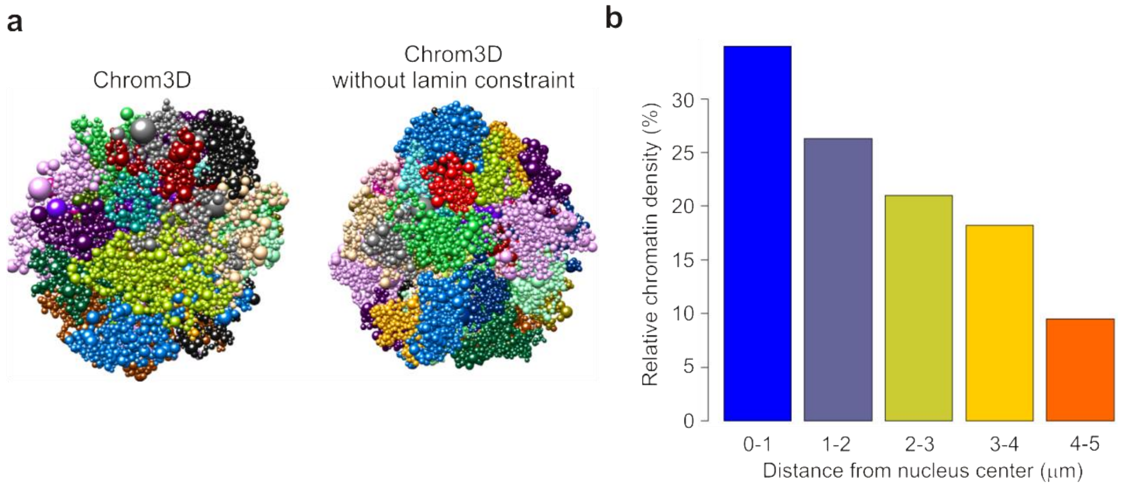

Fig. S4 Structure characteristics. **a** Examples of 3D genome structures modeled using Chrom3D (Hi-C and LMNA ChIP-seq data used as constraints) and using Chrom3D without the LMNA ChIP-seq constraint. **b** Relative chromatin density determined from 400 structures, in consecutive 1 µm-thick shells throughout the modeled nuclei. Chromatin density is defined as the ratio of (total bead volume in the shell / volume of the shell) and expressed as percentage.

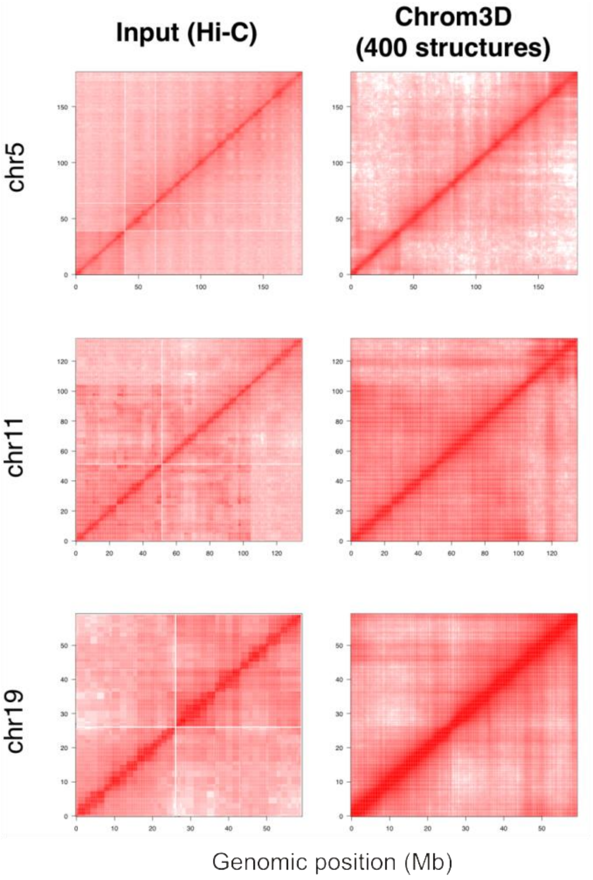

Chromosome	Correlation
chr1	0.9149703
chr2	0.9110089
chr3	0.9195702
chr4	0.8957786
chr5	0.9171892
chr6	0.9159752
chr7	0.9107025
chr8	0.9160578
chr9	0.9148295
chr10	0.9078822
chr11	0.9065500
chr12	0.9183737
chr13	0.8922376
chr14	0.9034258
chr15	0.9075271
chr16	0.8798919
chr17	0.9065312
chr18	0.8982765
chr19	0.8996001
chr20	0.9191211
chr21	0.8856351
chr22	0.8757341
chrX	0.8936033

Fig. S5 Correlation analysis between interaction frequency matrices reconstructed from 400 modeled structures and matrices from input Hi-C data. Examples of matrices are shown for three chromosomes. Correlations are shown for all chromosomes.

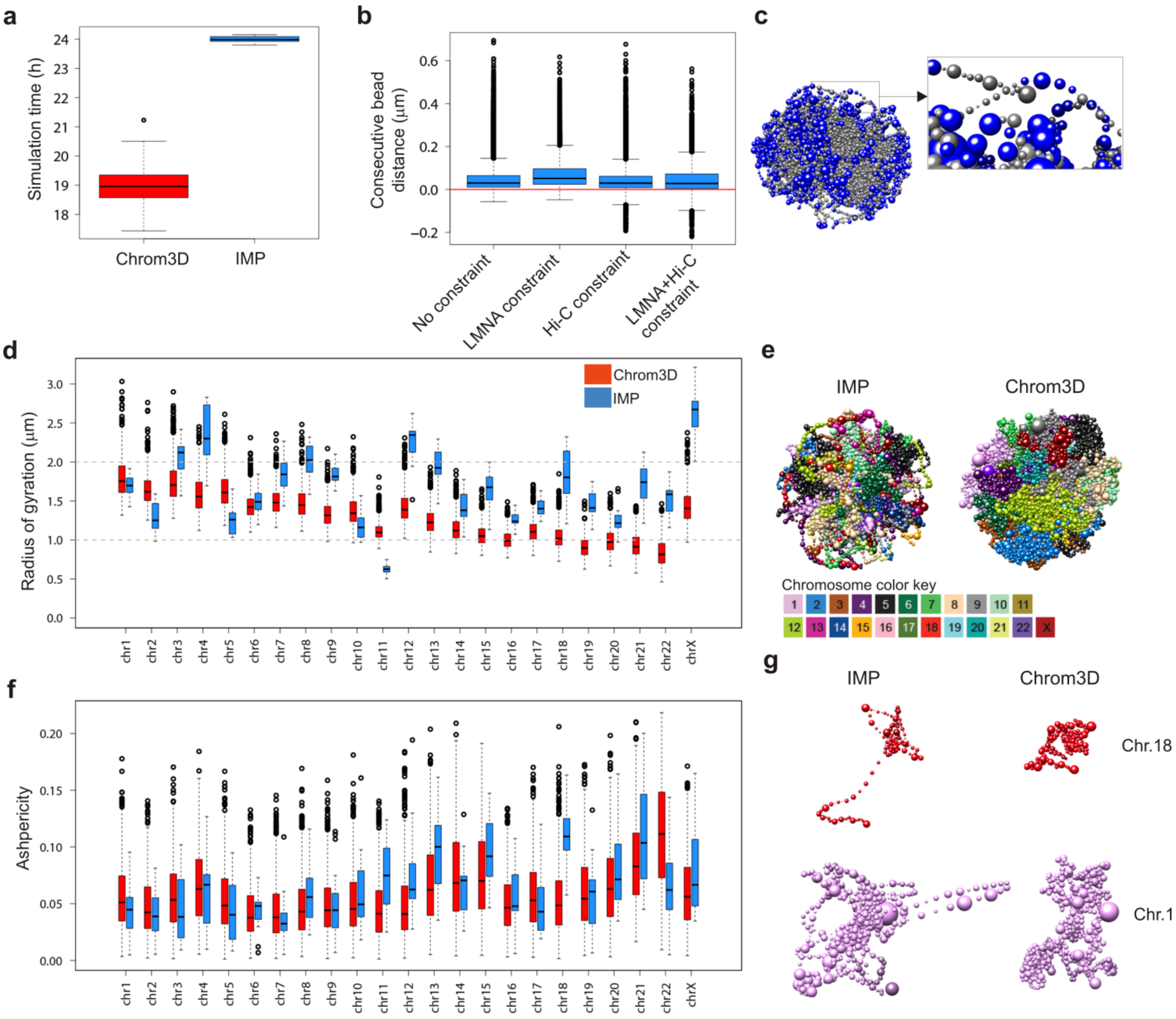

Fig. S6 Chrom3D and IMP comparison. **a** Total simulation time to convergence for 400 structures using Chrom3D and IMP. **b** Consecutive inter-bead distance in chromosome polymer chains for indicated IMP modeling constraints. Distance is measured by the Euclidean distance between two bead centers minus bead radii. Inter-bead distance with Chrom3D is by design zero regardless of constraint (red line). **c** An IMP 3D structure showing increased inter-bead distance upon constraining beads to the NP (blue beads represent A-TADs). **d** Radius of gyration (reflecting chromosome area) of each chromosome across 400 structures generated with Chrom3D, and 10 structures with IMP. **e** Chrom3D and IMP structures highlighting individual chromosomes. **f** Asphericity of chromosomes modeled by Chrom3D and IMP. **g** Asphericity shown for chromosomes 18 and 1. IMP code is available at http://folk.uio.no/jonaspau/imp-lamin.

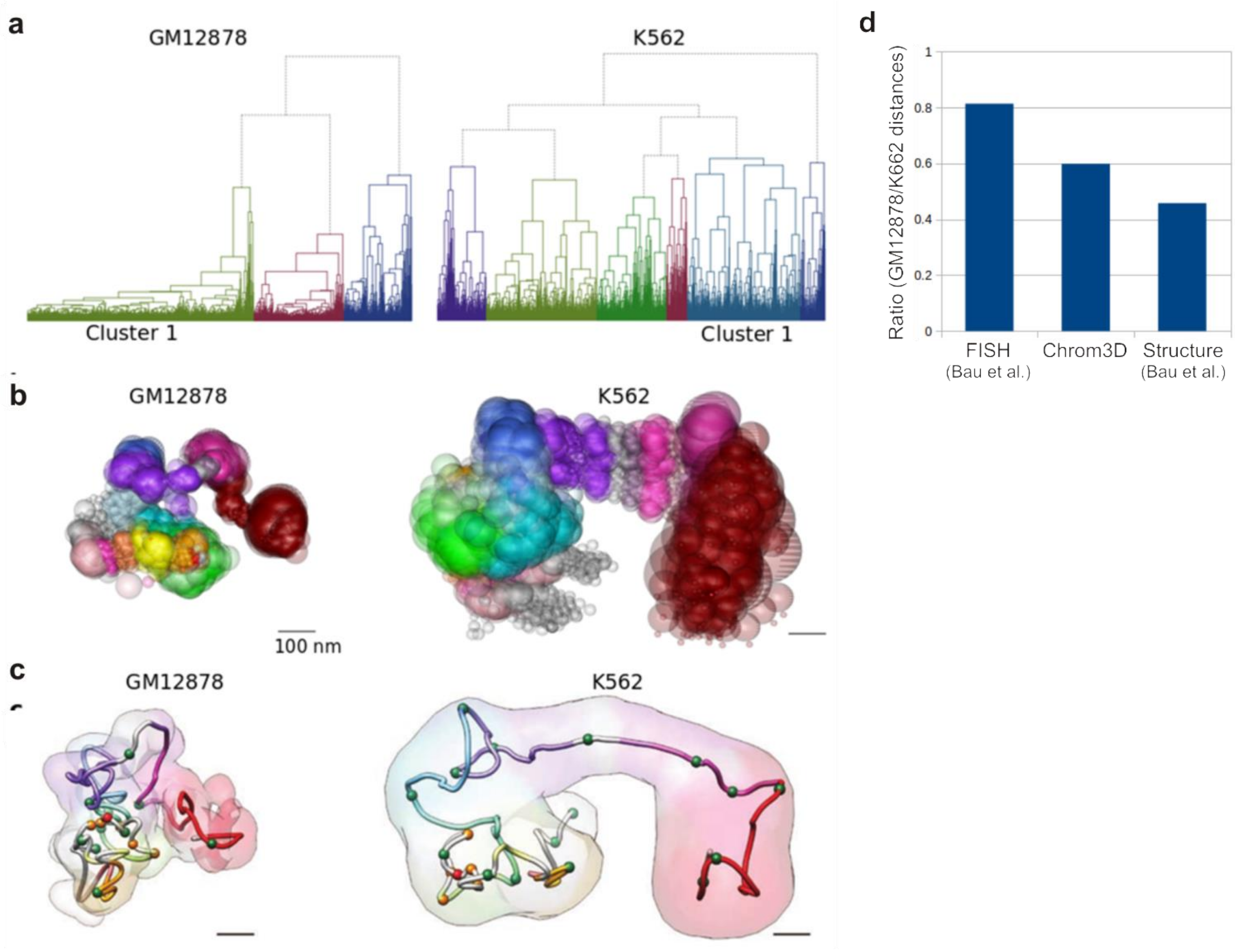

Fig. S7 Chrom3D modeling of the ENm008 ENCODE region containing the α-globin locus. **a** Clustering of 1,000 Chrom3D structures inferred from 5C data from Bau et al. [13] in GM12878 and K562 cells, in which the α-globin gene is respectively repressed and expressed. Note the range of structural clusters in K562 cells. **b** Chrom3D structures of the α-globin locus. Structures are shown as all structures from 'Cluster 1' for each cell type (672 and 314 structures for GM12878 and K562 respectively), superimposed with high transparency. The distance between the two ends of the modeled region (beads at positions 55911-56690 and 402437-418222 on chromosome 16) is greater in K562 than in GM12878, consistent with previously published structures. **c** Structures from Bau et al. [18] (reproduced with permission). In **b** and **c**, scale bar is 100 nm. **d** Ratios of distance between two end-beads in structures modeled by Chrom3D and by Bau et al [18] between GM12878 and K562 cells. Distance ratios for FISH probes from Bau et al. are also plotted.

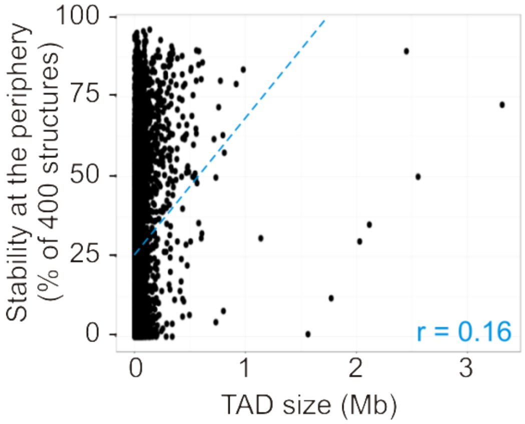

Fig. S8 Stability of TADs at the nuclear periphery as a function of TAD size, across 400 structures. Nuclear periphery is defined as in Fig. 2a. Note the lack of correlation, indicating that stability of placement of TADs at the NP across structures (Fig. 3b) is not merely influenced by TAD size.

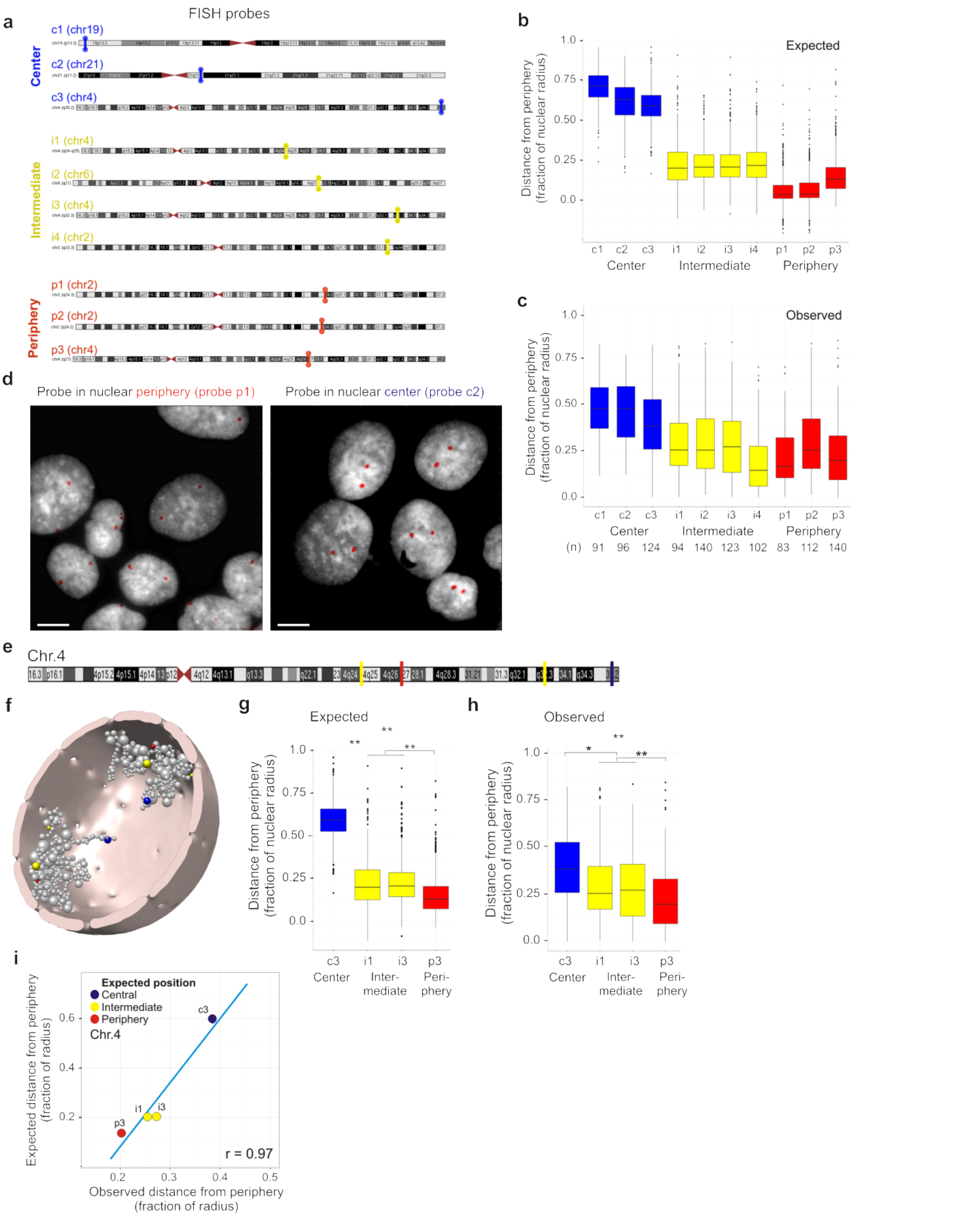

Fig. S9 FISH validation of radial LAD placement predicted by Chrom3D. **a** Genomic position of FISH probes. **b** Expected radial distribution of FISH probe across 400 structures. **c** Observed distribution

of FISH signals. n, numbers FISH signals analyzed. **d** Representative FISH images. Bars, 10 µm. **e** Position of FISH probes on Chr.4. **f** Representative structure showing spatial distribution of FISH probes on Chr.4. **g** Expected radial distribution of Chr.4 FISH probes across 400 structures. $**P < 2.2x10^{-16}$ (Mann-Whitney U test). **h** Observed radial distribution of a 453 FISH signals on Chr.4. $*P < 10^{-3}$; $**P < 10^{-4}$ (Mann-Whitney U tests). **i** Correlation between observed and expected relative distance of FISH signals to the nuclear periphery (chromosome 4).

a

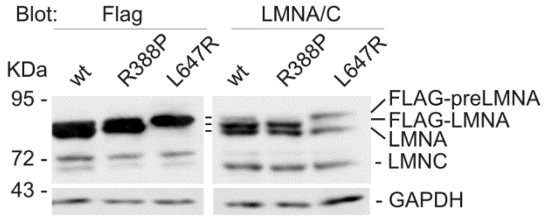

b

	No. peaks	Coverage (kb)	No. genes	Genes/Mb
**Flag constructs**				
LMNA (wt)	286	604 666	2848	4.71
LMNA(L647R)	290	621 860	2825	4.54
LMNA(R388P)	616	658 670	10936	16.60
**LMNA endogenous**	395	587 230	1312	2.23

c

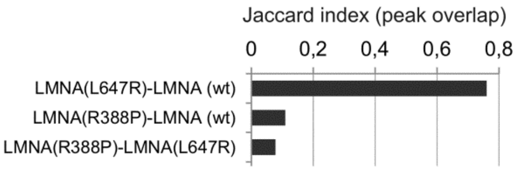

d

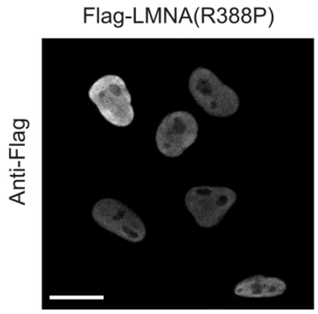

Fig. S10 Expression of Flag-LMNA wt, Flag-LMNA(R388P) and Flag-LMNA(L647R) in HeLa cells. **a** Western blot analysis of expression of each LMNA construct, using anti-Flag and anti-lamin A/C antibodies. GAPDH was immunoblotted as loading control. **b** Genomic properties of LADs for LMNA wt, LMNA(R647R) and LMNA(R388P) mapped by Flag ChIP-seq. Properties of endogenous LMNA LADs are also shown. **c** Jaccard index of overlap of LMNA wt and mutant LADs. **d** Immunofluorescence localization of Flag-LMNA(R388P); nucleoplasmic distribution is independent of expression level. Bar, 20 µm.

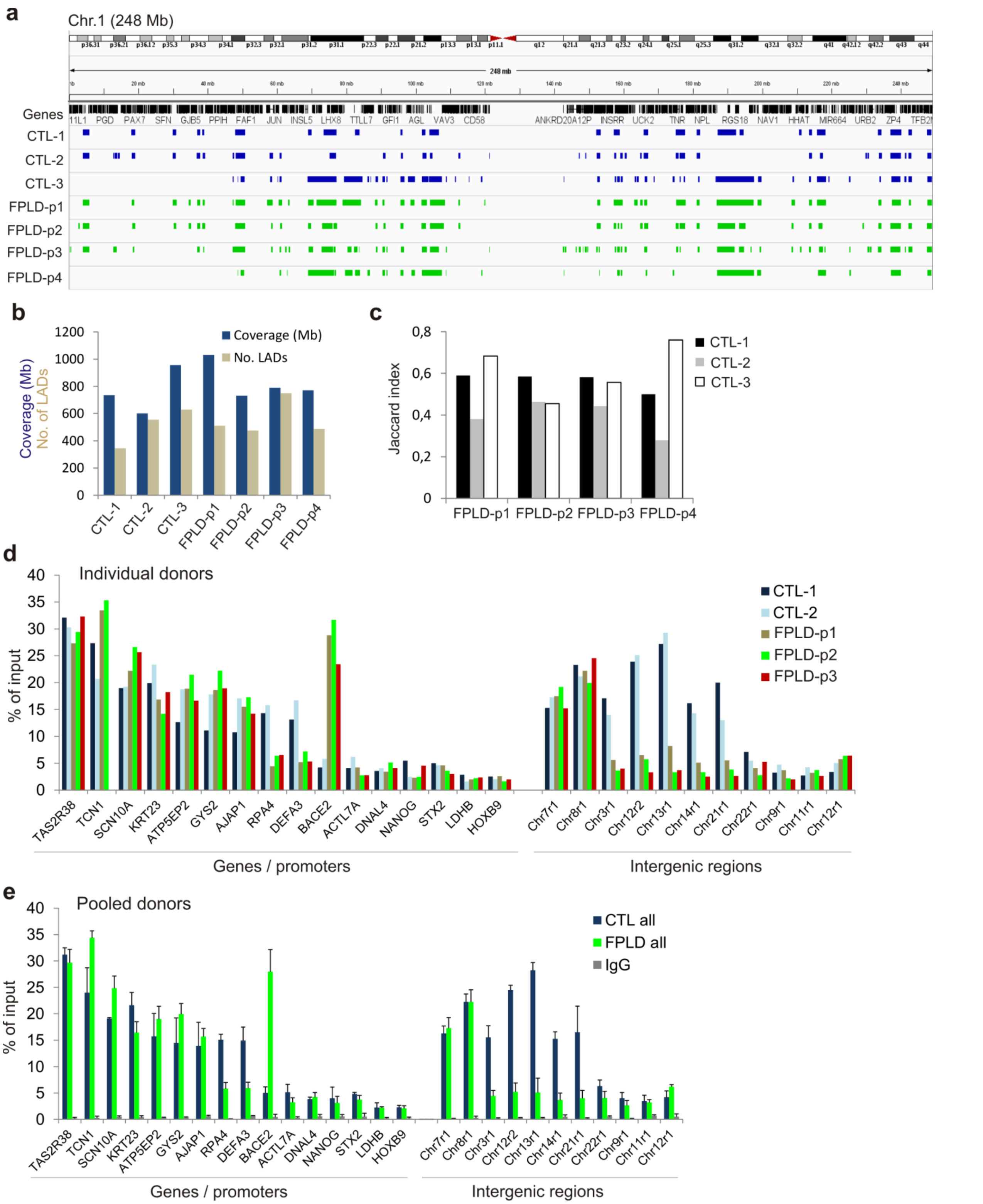

Fig. S11 LMNA LADs in FPLD2 patient and control fibroblasts. **a** IGV browser view (chromosome 1) of LADs for 3 controls (CTL) and 4 FPLD2 patients, mapped by LMNA ChIP-seq. Tracks for CTL-3 and all FPLD2 samples are new to this study. Tracks for CTL-1 and CTL-2 are generated from our previous data [46] (GEO GSE54334, samples GSM1313339, GSM 1313397). **b** LAD coverage and

number in each cell type, determined using Enriched Domain Detector [46]. **c** Jaccard index analysis of overlap of LADs in each of the four FPLD2 fibroblasts (x axis) with LADs of the three controls. **d** ChIP-qPCR validation of LMNA enrichment in indicated regions in control and FPLD2 fibroblasts. **e** Same data as in **D** but pooled within controls and patients. Control IgG ChIPs were for two control and two FPLD2 fibroblast cultures, and data pooled (mean ± SD of all donors).
